# Simultaneous Deletion of Virulence Factors and Insertion of Antigens into the Infectious Laryngotracheitis Virus Using NHEJ-CRISPR/Cas9 and Cre–Lox System for Construction of a Stable Vaccine Vector

**DOI:** 10.3390/vaccines7040207

**Published:** 2019-12-05

**Authors:** Mustafa Ozan Atasoy, Mohammed A. Rohaim, Muhammad Munir

**Affiliations:** Division of Biomedical and Life Sciences, Faculty of Health and Medicine, Lancaster University, Lancaster LA1 4YG, UK; m.atasoy@lancaster.ac.uk (M.O.A.); m.a.rohaim@lancaster.ac.uk (M.A.R.)

**Keywords:** ILTV, vaccine, virus control, genome editing, Cre–lox, recombinant vaccines

## Abstract

Infectious laryngotracheitis virus (ILTV) is a promising vaccine vector due to its heterologous gene accommodation capabilities, low pathogenicity, and potential to induce cellular and humoral arms of immunity. Owing to these characteristics, different gene-deletion versions of ILTVs have been successfully deployed as a vector platform for the development of recombinant vaccines against multiple avian viruses using conventional recombination methods, which are tedious, time-demanding, and error-prone. Here, we applied a versatile, and customisable clustered regularly interspaced short palindromic repeats (CRISPR)/Cas9 accompanied with Cre–Lox system to simultaneously delete virulence factors and to insert foreign genes in the ILTV genome. Using this pipeline, we successfully deleted thymidine kinase (*TK)* and unique short 4 (*US4*) genes and inserted fusion (*F*) gene of the Newcastle disease virus without adversely affecting ILTV replication and expression of the F protein. Taken together, the proposed approach offers novel tools to attenuate (by deletion of virulence factor) and to generate multivalent (by insertion of heterologous genes) vaccine vectors to protect chickens against pathogens of poultry and public health importance.

## 1. Introduction

Infectious laryngotracheitis virus (ILTV), also known as *Gallid herpesvirus 1*, causes economically devastating respiratory disease in chickens around the globe. ILTV is a prototypic member of the genus *Iltovirus* of the family *Herpesviridae* and primarily replicates in the epithelial cells of the upper respiratory tract, including conjunctiva, trachea, and larynx [1], leading to the characteristics gasping, coughing, expectoration of bloody mucus, and conjunctivitis. Generally, ILTV remains latent in sensory neurons without any apparent clinical signs; however, due to various stresses, occasional reactivation can lead to virus shedding [2].

Similar to mammalian herpesviruses, the genome of ILTV carries a number of unique long (UL) and unique short (US) sequences. However, the genomic organisation and genetic distances are distinctive for ILTV [3,4]. Compared to other herpesviruses, iltovirus-specific genes include relocation of *UL47* gene from UL to US regions, the inversion of a conserved gene cluster within the UL region (i.e., *UL22* to *UL44*) [5,6,7]. The ILTV carries 12 glycoproteins, and several of these proteins (e.g., *gB, gC, gG, gJ, gM,* and *gN*) have been functionally characterised. On the other hand, several glycoproteins (*gG, gJ, gM,* and *gN*) are not crucial for virus replication, and deletion of *gG* and *gJ* leads to the attenuation of ILTV in chickens [8,9]. The *UL23* (encoding for thymidine kinase, *TK*) is one of the well-characterised virulence factors in herpesviruses, and deletion of *TK* gene attenuates the pathogenicity of ILTV, while maintaining the immunogenicity against challenged virus [10]. The *US4* (*gG*) is a non-structural protein (not incorporated into mature virus particles) and is secreted from infected cells only [11,12]. Based on studies on multiple herpesviruses, it has been proposed that *US4* modulates the host immune response by binding to chemokines [13], and *gG*-negative bovine herpesvirus-1 (BHV-1) mutants were attenuated in cattle [14]. Taken together, knocking out *TK* and *US4* from ILTV genome could propose a stable vaccine vector. 

Owing to low mortality and moderate impacts on weight gain and egg production, the live attenuated strains of ILTV are widely exploited as a vector to develop recombinant and multivalent vaccines [15,16,17]. Moreover, due to the replication in the upper respiratory tract, the vaccine can successfully be deployed for mass application through eye-drops, aerosol, or drinking water [18]. The ILTV vector proposes additional advantages through potent induction of both cellular and humoral immune responses, and enabling differentiation between infected and vaccinated animals (DIVA) [18]. In both of these applications, conventionally attenuated live-virus vaccines are applied, which can pose significant residual virulence or can potentially revert into virulent phenotypes after several passages in animals [19,20,21]. To propose a safer vectored vaccine, efforts have been made to delete virulence genes from the ILTV genome, either by conventional homologous recombination in virus-infected cells or through recombineering techniques on full-length genomes [18]. However, the generation of recombinant ILTV using these methods is time-consuming, requiring the construction of transfer vectors and several rounds of plaque purifications to obtain the recombinant vaccine candidate. These shortcomings warrant application of next generation genome-editing approaches for the generation of safer, stable, and efficient vaccine vectors to be deployed in avian disease (e.g., Newcastle disease virus (NDV), avian influenza, and infectious bronchitis virus) control programmes. 

The recent discovery of CRISPR (clustered regularly interspaced short palindromic repeat)–Cas9 system has revolutionised genome editing and is opening new avenues of genetic manipulation. The functional components of type II CRISPR–Cas system include the RNA-guided Cas9 endonuclease, originally characterised in multiple bacterial species such as *Streptococcus pyogenes*, a single guide RNA (sgRNA), and the trans-activating crRNA (tracrRNA) [22,23]. The sgRNA direct the Cas9 protein to the 20 nucleotide target sequence adjacent to a 5′-NGG-’3 protospacer adjacent motif (PAM). Upon binding, the Cas9 endonuclease introduces a double strand break (DSB) in this target sequence. The DSBs can then be repaired by either an error-prone non-homologous end-joining (NHEJ) or the high-fidelity homology-directed repair (HDR) pathway. This approach has been applied for genome editing of mammalian cells [23], for genetic modification in animal models [24], and genomic manipulation of several DNA viruses such as herpes simplex virus type I, adenovirus, pseudorabies virus, vaccinia virus, Epstein–Barr virus, guinea pig cytomegalovirus, herpesvirus of turkey, and duck enteritis virus [25,26,27,28,29,30,31,32]. Accompanied by CRISPR/Cas9, the Cre–Lox recombination system can be applied to excise a pre-defined LoxP sites (34 base-pair DNA sequence) to cleave the DNA surrounded by LoxP sites [33].

In this study, we developed and applied a highly efficient, versatile, and rapid NHEJ-CRISPR/Cas9 and Cre–Lox-mediated genome-editing approach for simultaneous deletion of pre-determined virulence factors and insertion of viral antigen to generate recombinant, multivalent, and safer vaccine vectors. We demonstrated the use of this approach first by the generation of a reporter virus and deletion of *TK* gene from ILTV genome. Thereafter, we engineered an ILTV-vectored vaccine candidate harbouring fusion gene (*F*) of the velogenic NDV and deletion of another gene, *US4*, from the ILTV genome. The reporter marker gene was excised using Cre–Lox system without affecting the expression and stability of the F protein. This potential vaccine candidate was assessed for the stable expression of inserted protein, replication kinetics, and for comparative *in vitro* characteristics. We demonstrate that NHEJ-CRISPR/Cas9 accompanied by Cre–Lox is an efficient method for rapid generation of ILTV-based recombinant vaccines and proposes multiple advantages to traditional recombination and recombineering techniques

## 2. Materials and Methods 

### 2.1. Cell Culture and Viruses

Leghorn male hepatoma (LMH) cells, kindly provided by Prof. Venugopal Nair, The Pirbright Institute UK, were maintained in Dulbecco’s Modified Eagle Medium (DMEM; Sigma-Aldrich, St. Louis, MO, USA) supplemented with 10% foetal bovine serum GlutaMAX and penicillin–streptomycin (100 U/mL, all from Thermo Fischer Scientific). *Gallid alphaherpesvirus 1 strain* was obtained from American Type Culture Collection.

### 2.2. Construction of sgRNAs and Donor Plasmids

The gRNA targeting the UL47/US4 intergenic region and downstream of *US4* gene in ILTV genome was designed using online CRISPR guide RNA designing software (http://crispr.mit.edu/). Synthesised oligonucleotide pairs were annealed and cloned into BbsI restriction sites of pX459-v2 CRISPR/Cas9 vector (Addgene #62988). For the construction of green fluorescent protein (GFP) donor cassette, the GFP was flanked upstream with pEFa promoter and downstream with the polyA tail. Synthetic oligos carrying sgA target sites were cloned on 5′ and 3′ ends of this cassette (Table S1). For the construction of the dsRED carrying donor plasmid (pCDNA3-CRISPR-Lox-wt), a synthesised oligo containing two multiple cloning sites, a pair of LoxP sites, and two guide RNA targeting sequences (SgA and SgB) were digested with BsmBI and cloned into pCDNA3 vector via BbsI and MfeI restriction sites. The *dsRED* gene was amplified from pCDNA3-dsRED vector and cloned into MCS2 of donor backbone with the use of EcoRI and XbaI enzymes, generating pCDNA3-CRISPR-Lox-MCS2dsRED. Finally, the *F* gene of NDV was amplified from genotype VII strain of Egyptian origin with FClon1F/R primers set and cloned into MCS1 site by NheI and KpnI double digestion to generate pCDNA3-CRISPR-Lox-MCS1F-MCS2dsRED.

### 2.3. Generation of Recombinant ILTV

LMH cells were seeded in 6-well plates and a total of 1 µg of plasmid expressing each of the gRNAs, along with 1 µg donor plasmid, were co-transfected into LMH cells using Lipofectamine 2000 (Thermo-Fischer Scientific) according to the manufacturers’ instructions. At 24 h post-transfection, ILTV was infected into the LMH cells at 0.01 multiplicity of infection (MOI). Cells were incubated until plaques progressively appeared for GFP or both GFP and dsRED signals. Those plaques were then picked and passaged several times in LMH cells and confirmed by PCR. 

Cre–Lox system was used to excise dsRED from recombinant virus backbone. Briefly, 6-well plates including LMH cells were transfected with 2 µg of the pCAG-Cre (Addgene #13775). After 24 h post-transfection, cells were infected with 100 plague-forming units of ΔTK&ΔUS4-dsRED+ve ILTV. Cells were again incubated until only GFP appeared in cells. These plagues were then picked and confirmed by PCR and confocal microscopy.

### 2.4. Characterisation of Recombinant ILTV Viruses

LMH cells were plated in 6-well plates for 24 h before infection with purified ΔTK-GFP+ ILTV, ΔTK&ΔUS4-dsRED+ve ILTV, ΔTK&ΔUS4-Cre ILTV or wild type (wt) ILTV the following day. The infected cells were harvested after 72 h of infection and subjected for DNA extraction using a DNeasy Blood & Tissue Kit (Qiagen). PCR targeting the *F* gene was carried out using specific primers, shown in Table S1, for identification of the recombinant ΔTK&ΔUS4-dsRED+ve ILTV compared to ILTV wild-type and ΔTK-GFP+ ILTV. The PCR products were purified using a GenElute Gel Extraction Kit (Sigma Aldrich, Germany) and sequenced using the primers listed in Table S1 to confirm the insertion of the *F* gene.

### 2.5. Western Blot Analysis

Expression of the F protein in recombinant virus-infected LMH cells was determined by Western blot analysis using anti-F monoclonal antibody (Mab) [34] as the primary antibody. After 48–72 h post-infection with recombinant viruses, cells were washed one time with ice-cold phosphate buffered saline (PBS). Lysis for the infected cells was carried out by adding 100 μL ice-cold NP40 buffer (completed with protease inhibitors cocktail) per well for 30 min on ice. Cells were scrapped off and transferred to 1.5 mL microfuge tube. Using 25 G needle X6, the lysate was passed through the up and down to ensure complete disruption of cells. The lysis was then left on ice for 15 min, followed by centrifugation at 13,000 rpm for 5 min at 4 °C. The lysates (supernatant) of infected cells were incubated in sample buffer containing 10% β-mercaptoethanol for 5 min at 98 °C, and separated on a 10% sodium dodecyl sulfate–polyacrylamide gel electrophoresis (SDS-PAGE), and the resolved proteins were transferred onto polyvinylidene difluoride (PVDF) membranes. Immunoblots were blocked with 5% skimmed milk, and then incubated with primary antibodies (1:100 dilution). After probing with primary antibodies, the blots were incubated with peroxidase-conjugated species-specific secondary antibodies and visualised by chemiluminescence (Chemidoc, BioRad, Hercules, CA, USA).

### 2.6. Indirect Immunofluorescence Analysis

The expression of the F protein in recombinant virus-infected cells was evaluated by immunofluorescence assays using confocal microscopy. LMH cells, grown on coverslips in 24-well plates, were infected with the recombinant viruses for 48–72 h before harvesting. After fixing the cells with 4% paraformaldehyde and permeabilisation with 0.1% Triton X-100, cells were incubated with primary antibodies raised against NDV [34]. Afterwards, cells were incubated with corresponding secondary antibodies for 2 h. The F protein expression was analysed through fluorescence for wild-type and recombinant ILTV. Cell nuclei were then stained with 40,6-diamidino-2-phenylindole (DAPI), and the images were taken using a Zeiss confocal laser-scanning microscope (Zeiss, Kohen, Germany).

### 2.7. Stability of the Inserted Genes in the Recombinant ILT Viruses

The recombinant viruses were grown sequentially in LMH cells for at least 15 passages. The integrity of the *F* gene was examined by PCR (with primer pairs FusClon1 and FusClon2 which amplifies the full-length *F* gene) using DNA extracted from different passages.

### 2.8. In Vitro Growth Kinetics and Plague Assay

To investigate the growth properties of the recombinant ILTV, LMH cells in were infected in 6-well plates with 100 pfu of each virus (wild-type ILTV, ΔTK-GFP+ ILTV, and ΔTK&ΔUS4-dsRED+ve ILTV) and harvested at 0, 4, 8, 12, 24, 48, and 72 h post-infection to determine the *in vitro* growth kinetics using the methods described previously [35]. DNA was extracted using a DNeasy Blood & Tissue Kit (Qiagen) and used for real-time qPCR. Real-time qPCR was carried out to detect ILTV *gB* gene [36], which enabled the calculation of ILTV genome copies that was plotted against hours post-infection for each of the viruses to produce standard curves.

LMH cells were infected at MOI of 0.2, and the growth kinetics were assayed as described [37,38]. Progeny virus plaques were visualised by IFA using an NDV F-specific MAb [34] or fluorescence for ΔTK-GFP+ ILTV and ΔTK&ΔUS4-dsRED+ve ILTV. At different time points after infection, cells and supernatants were harvested and lysed by freeze–thawing and the mean progeny titres for each time point after infection were calculated. Furthermore, 30 plaques per virus mutant were measured microscopically, and average diameters as well as standard deviations were calculated.

### 2.9. Statistical Analyses

Pairwise comparisons of virus-treated and control groups were performed using Student’s *t*-test. All statistical analyses and figures were conducted in the GraphPad Prism (GraphPad Software, La Jolla, CA, USA).

## 3. Results

### 3.1. Targeted Deletion of TK Gene and Simultaneous Knock-In of GFP Expression Cassette into the ILTV Genome Using NHEJ-CRISPR/Cas9 Approach

**Figure 1:**
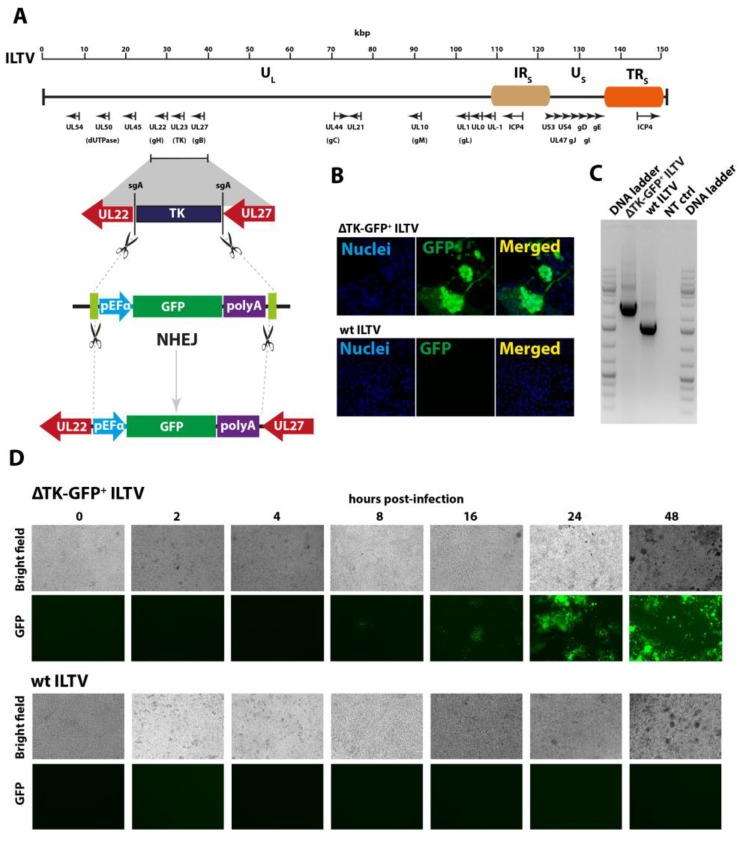
Homology-independent knocking-out of *TK* gene and knocking-in of a reporter gene into the infectious laryngotracheitis virus (ILTV) genome using CRISPR/Cas9-induced and non-homologous end-joining (NHEJ)-mediated double stranded DNA break and repair mechanisms. (**A**) Schematic diagram outlining the genomic organisation of the ILTV genome. The internal and terminal repeat short (IRS/TRS) and unique long (UL) and short (US) regions of the ILTV genome are shown. Important viral genes and their orientations are labelled at the bottom and approximate scale of the genome size is displayed at the top of the viral annotated genome. The schematic illustration of the donor plasmid and targeting strategy for CRISPR/Cas9-induced and NHEJ-mediated insertion of green fluorescent protein (GFP) reporter gene, which replaces the *TK* gene in the ILTV genome. The upstream promoter, downstream polyA tail, and sgRNA sites flanking the expression cassette are shown. (**B**) The leghorn male hepatoma (LMH) cells were transfected with plasmid carrying donor reporter cassette, expressing sg-A and Cas9 for 24 h and infection with ILTV until appearance of foci. Immunofluorescence visualisation of the ILTV plagues expressing GFP cassette (upper panel) and wild type (wt) ILTV control (lower panel). (**C**) LMH cells were infected with ΔTK-GFP+ ILTV, wild-type ILTV, or left uninfected for 48 h before extraction of the total DNA for PCR analysis. (**D**) LMH cells were infected with ΔTK-GFP+ ILTV and wt ILTV, and the expression of the GFP was visualised using fluorescence microscopy at different time points for 2 days.

The thymidine kinase (TK) is one of the most virulent factors in ILTV pathogenesis, and it has been previously reported that deletion of *TK* from the ILTV genome attenuates the virus without compromising the replication and immunogenicity [39]. To demonstrate the genome editing approach for ILTV, we first aimed to delete the *TK* gene by CRISPR–Cas9 excision using target-specific sgRNA at both up- and downstream regions of the *TK* gene (Table S1). In order to exclude any possible deleterious effects of this deletion on the virus replication, and to simultaneously generate a marker virus, a reporter donor plasmid was constructed. This plasmid carried GFP reporter gene cassette comprised of eukaryotic translation elongation factor alpha 1 (pEFα) promoter, gene encoding GFP and corresponding polyA tail. The entire cassette was flanked with sgRNA-targeted sites to cleave the GFP expression fragment *in vitro* (Figure 1A). Two sgRNA (sgA) corresponding the targeting sites in the donor plasmid to release expression fragment and two sgRNA targeting the viral genome surrounding the *TK* gene were cloned into pX459v2, which expresses sgRNA by the U6 promoter and bicistronically expresses the human codon optimised *Streptococcus pyogenes* Cas9 through cytomegalovirus (CMV) promoter. Cleavage of the viral genome by Cas9 and concurrent excision of expression cassette from the donor plasmid DNA will simultaneously delete the *TK* gene and introduce the GFP, as outlined in Figure 1A.

The NHEJ is a superior pathway over HDR and has been demonstrated by several studies in eukaryotic cells [28], animals [24], and DNA viruses [31]. Therefore, we used NHEJ mechanisms to knock-in the expression cassettes into the viral genome. To demonstrate this process for the ILTV, the CRISPR/Cas9 system, including donor plasmid, sgRNAs targeting ILTV genome, and sgRNAs cleaving the donor plasmid, were transfected in LMH cells for 24 h and were infected with multiplicity of infection (MOI, 1.0) of wild-type ILTV. These transfected and subsequently infected cells were passaged after every 3 days until GFP plagues were observed (Figure 1B). These progeny virus plaques carrying the knock-in cassette of GFP and deleted *TK* genes were individually picked, propagated, and designated as ΔTK-GFP^+^ ILTV.

In order to confirm the insertion of the GFP in the TK locus, we amplified the *TK* gene-flanking region using site-specific primers (Table S1). Compared to the wild-type ILTV, the ΔTK-GFP^+^ ILTV showed an insert of GFP (Figure 1C). However, this insertion could be due to DSB by a single gRNA instead of *TK* excision. To exclude this possibility, we sequenced the amplified PCR products and observed an excision of intentional *TK* gene and insertion of the GFP reporter gene, highlighting the occurrence of InDel events and error-prone NHEJ-mediated DNA repair mechanism.

Next, we demonstrated the replication kinetics of ΔTK-GFP^+^ ILTV and showed that ΔTK-GFP^+^ ILTV replicated competitively and progressively (Figure 1D) over a period of 2 days. The purified virus was passaged for more than 25 times without the repulsion of the marker GFP, demonstrating the stability of the insert (see below). These data validate the proposed approach on simultaneous deletion and insertion of genes in the ILTV genome and potential for the development of safer and stable vaccine candidates.

### 3.2. Deletion of US4 and Targeted Knock-In of Fusion Gene of NDV and dsRED Expression Cassettes into the ΔTK-GFP+ILTV Genome

**Figure 2:**
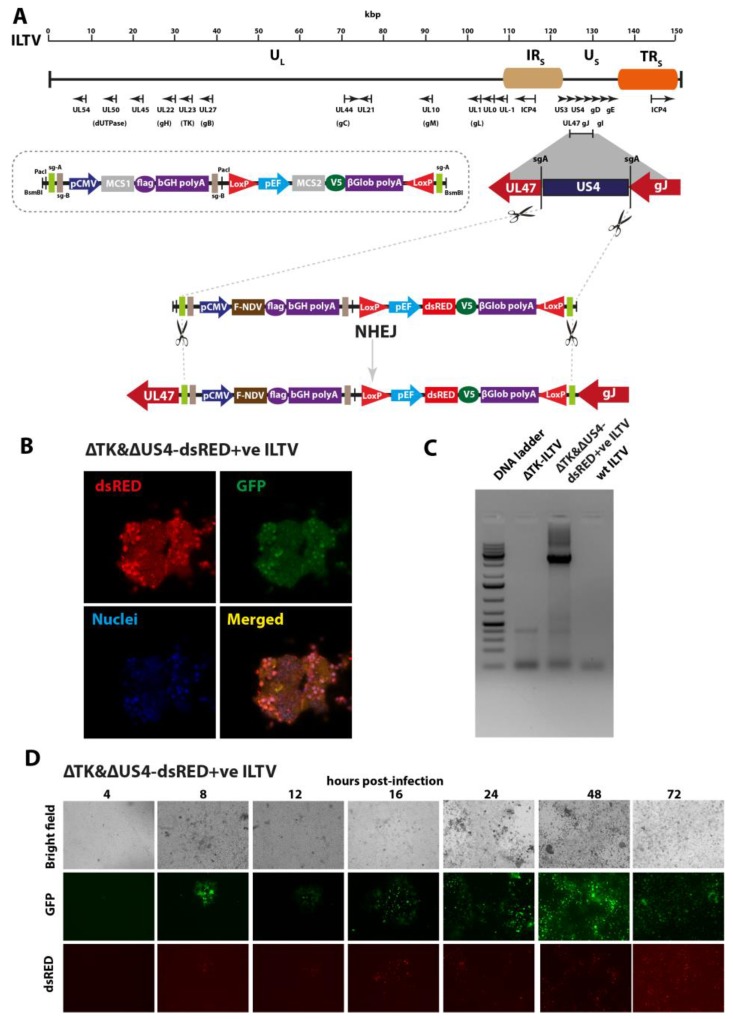
Homology-independent knock-out of *US4* and knock-in of the *F* gene of Newcastle disease virus (NDV) into the ILTV genome using CRISPR/Cas9. (**A**) Schema of the ILTV and strategy for the construction of donor plasmid. The insertion cassette is shown in the inset. Two multiple cloning sites (MCS) were flanked upstream with the promoter and downstream with the corresponding polyA tail. Full length *F* gene of the NDV was cloned in the MCS1 site whereas dsRED was cloned in the MCS2 site. The dsRED cassette was flanked with the LoxP sites at the 5′ and 3′ ends to be excised by Cre-recombinase. The entire cassette was flanked with sgA/sgB target site to release the cassette by the Cas9 and was cloned between BsmB1 sites in the pcDNA3.1. The donor plasmid and plasmids expressing sgRNA targeting the entire length of the US4 and cassette, as well as Cas9 expression plasmids, were co-transfected in LMH cells for 24 h before infection with ΔTK-GFP+ ILTV. (**B**) The plagues co-expressing the GFP (representing ΔTK-GFP+ ILTV) and dsRED (representing ΔTK&ΔUS4-dsRED+ve ILTV) were visualised by confocal microscopy. (**C**) LMH cells, infected with ΔTK-GFP+ ILTV, ΔTK&ΔUS4-dsRED+ve ILTV, and wt ILTV viruses, were collected for PCR analysis. (**D**) LMH cells were infected with ΔTK&ΔUS4-dsRED+ve ILTV, and the expression of the GFP and dsRED was visualised using fluorescence microscopy at different time points for 3 days.

After successful demonstration that marker genes could be inserted in the target site of the ILTV genome and the corresponding genes can be deleted from the backbone of ILTV by CRISPR/Cas9 technology, we aimed to construct a recombinant bivalent vaccine against NDV and ILTV. To further facilitate the efficient and rapid development of recombinant vaccines, we generated a universal and versatile donor system, which can be exploited to develop vaccine independent of pathogen or host sequences. The entire cassette consisting of expression of two individual genes by independent promoters was generated (Figure 2A, inset). The dsRED expression cassette was then flanked with LoxP sites to facilitate the excision of the marker gene by the Cre-recombinase (Figure 2A). Additionally, the full-length cassette and the antigen (*F* gene of NDV in this case) expression cassette were flanked with the sgA or sgB for *in vitro* swapping of corresponding genes, respectively.

In addition to *TK* deletion, we aimed to delete *US4*, which is a known immune modulator in ILTV infection, to further enhance the safety profile of the ILTV vaccine vectors. A total of three pairs of sgRNA targeting variable regions in and around the *US4* gene were cloned into the pX459v2, which expresses sgRNA via U6 promoter and Cas9 by the CMV promoter (Table S1).

The LMH cells were co-transfected with donor plasmid, as well as with Cas9/gRNA vectors targeting *US4* and donor vector cleavage sites for 24 h, and then were infected with ΔTK-GFP^+^ ILTV. These transfected/infected cells were passaged until dsRED plaques appeared and overlapped with the GFP plaques (Figure 2B). The progeny virus plaques expressing dsRED were isolated, plague purified and termed as ΔTK&ΔUS4-dsRED^+^ ILTV. In order to demonstrate the insertion of the entire cassette, the plague purified viruses were used to extract total genomic DNA and used to amplify the cassette using flanking 5′ and 3′ specific primers (Table S1). The product size and sequencing of the fragments showed the deletion of *US4* and insertion of the entire cassette of ~5 kb in size (Figure 2C). We also demonstrated that insertion of the expression cassette did not show prominent impacts on the replication kinetics of the ΔTK&ΔUS4-dsRED^+^ ILTV determined by the sequential fluorescent microscopy (Figure 2D) and qPCR targeting gB gene of the ILTV. Moreover, recombinant and wt ILTVs showed marked replication, as shown by plaque assays in LMH cell line.

### 3.3. Excision of the dsRED from Knocked-In Expression Cassette from the ΔTK&ΔUS4-dsRED+ ILTV Genome Using Cre–Lox Recombinase

**Figure 3:**
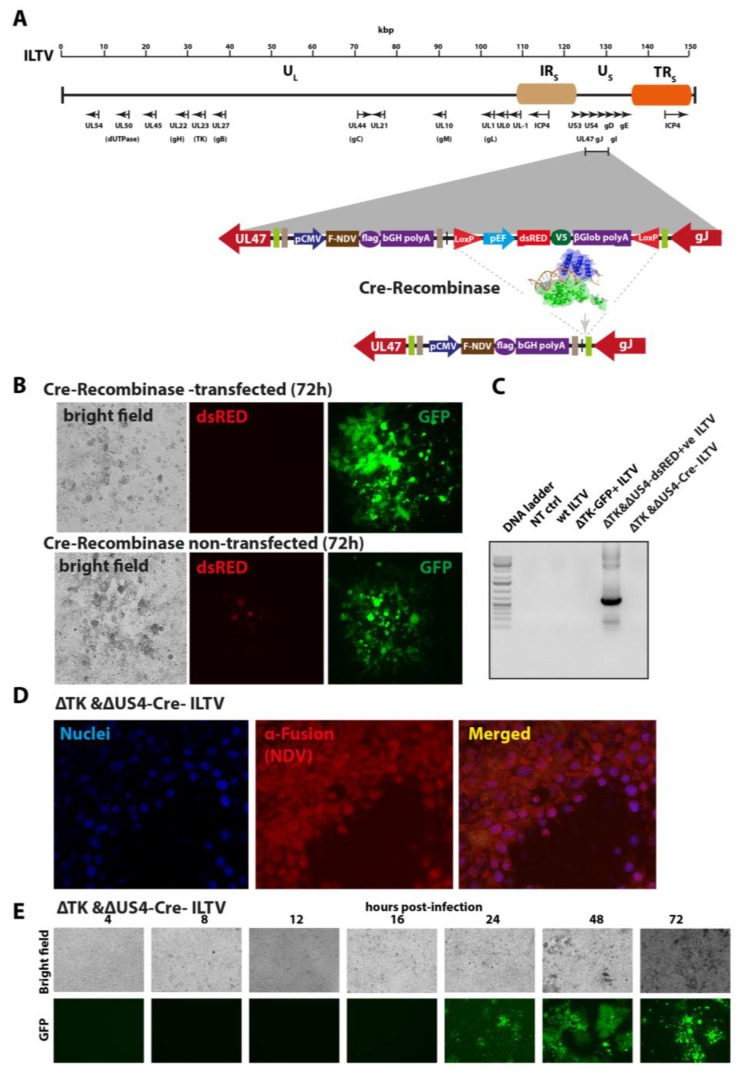
Excision of dsRED from the ΔTK&ΔUS4-dsRED+ve ILTV using Cre–Lox system. (**A**) Genomic organisation of the ILTV and strategy for the Cre-recombinase-mediated excision of the dsRED through LoxP sites. (**B**) LMH cells were transfected with Cre-recombinase expressing plasmid or mock transfected for 24 h and were then infected with ΔTK&ΔUS4-dsRED+ve ILTV for 72 h. The excision of the dsRED was observed using fluorescence microscopy. Bright field, and dsRED and GFP-positive fields were presented. (**C**) LMH cells infected with wt ILTV, ΔTK-GFP+ ILTV, ΔTK&ΔUS4-dsRED+ve ILTV, and ΔTK&ΔUS4-Cre-ILTV viruses were collected for PCR analysis. Note the absence of dsRED in ΔTK&ΔUS4-Cre-ILTV-infected cells. (**D**) LHM cells were infected with ΔTK&ΔUS4-Cre-ILTV and stained for the F protein of NDV. (**E**) LMH cells were infected with ΔTK&ΔUS4-Cre-ILTV, and the expression of the GFP was visualised using fluorescence microscopy at different time points for 3 days.

**Figure 4:**
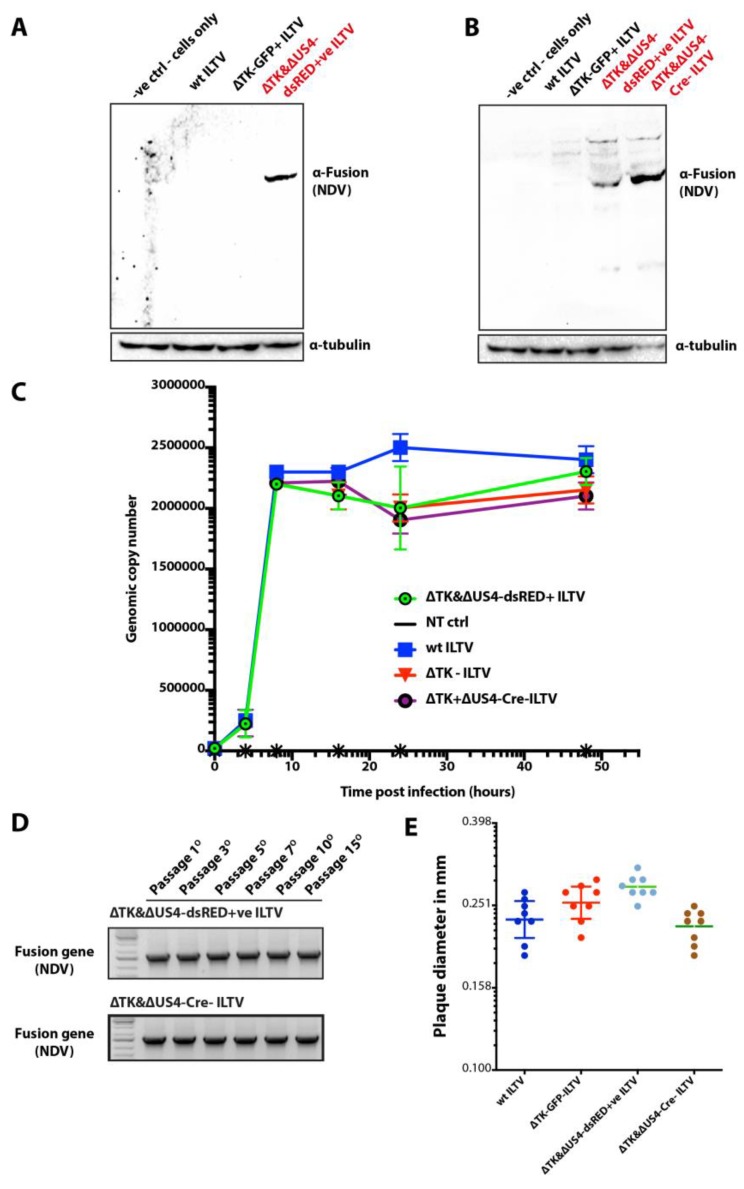
Characterisation of the recombinant ILTVs generated using CRISPR/Cas9 and Cre–Lox system. (**A**) LMH cells were infected with wt ILTV, ΔTK-GFP+ ILTV, or ΔTK&ΔUS4-dsRED+ve ILTV (all passaged at least 15 times) or left uninfected for 48 h. Cells were lysed and expression of the F protein of NDV was determined using Western blotting, and alpha tubulin was used as loading control. (**B**) LMH cells were infected with wt ILTV, ΔTK-GFP+ ILTV, ΔTK&ΔUS4-dsRED+ve ILTV, ΔTK&ΔUS4-Cre-ILTV, or left uninfected for 48 h. Cells were lysed and expression of the F protein of NDV was determined using Western blotting, and alpha tubulin was used as loading control. (**C**) *In vitro* growth kinetics were performed using real-time PCR on DNA extracted from LMH cells at various time points post-infection with wt ILTV, ΔTK-GFP+ ILTV, ΔTK&ΔUS4-dsRED+ve ILTV, ΔTK&ΔUS4-Cre-ILTV, or from uninfected cells. (**D**) PCR was applied to confirm the stability of *F* gene expression cassette from the recombinant viruses at passage 1, 3, 5, 7, 10, and 15 in LMH cells using primers targeting the *F* gene. (**E**) LMH cells were left uninfected or infected with wt ILTV, ΔTK-GFP+ ILTV, ΔTK&ΔUS4-dsRED+ve ILTV, or ΔTK&ΔUS4-Cre-ILTV for 2 h and were covered with overlay media for 10 days before staining with crystal violet and measuring for plaque sizes.

In order to delete the dsRED from ΔTK&ΔUS4-dsRED^+^ ILTV, LHM cells were transfected with Cre-recombinase expressing pcDNA3-Cre plasmid (Addgene#13775) for a day before infection with the recombinant virus (Figure 3A). Cells expressing GFP only where dsRED was excised by the recombinase (dsRED negative) were selected, purified, propagated, and designated as ΔTK&ΔUS4-Cre ILTV (Figure 3B). The deletion of dsRED was further confirmed in the purified recombinant virus by PCR primers specifically targeting the dsRED (Table S1). As expected, the dsRED expression cassette was only detected in the ΔTK&ΔUS4-dsRED^+^ ILTV, whereas the ΔTK&ΔUS4-Cre ILTV lacked the dsRED cassette (Figure 3C). Immunostaining of the F protein of NDV in ΔTK&ΔUS4-Cre-infected LMH cells showed an intact expression of transgene even after dsRED excision (Figure 3D). The dsRED-deleted ILTV replicated competitively compared to the ΔTK&ΔUS4-dsRED^+^ ILTV, as was observed in the time course fluorescence microscopy (Figure 3E), qPCR, and plaque assay (Figure 4). These results demonstrate that Cre–Lox system offers a convenient tool to eliminate the expression of selection genes, without affecting the expression of spared genes and virus replication.

### 3.4. Stable Expression of Fusion Protein of NDV in Recombinant ILTV

Having demonstrated that CRISPR/Cas9 and Cre–Lox system is an effective, convenient and fast approach to generate recombinant vaccine candidates and to generate potential attenuated strains, we next investigated if the inserted *F* gene of NDV could be expressed in recombinant ILTVs. For this purpose, LMH cells were either mock-treated or infected with wt ILTV, ΔTK-GFP^+^ ILTV or ΔTK&ΔUS4-dsRED^+^ ILTV, and the lysate was use for the expression of the F protein. The expression was assessed by Western blotting using antibodies against the F protein of the NDV whereas antibodies targeting alpha tubulin were used as loading control. As anticipated, cell lysate from ΔTK&ΔUS4-dsRED^+^ ILTV-infected cells demonstrated expression of the F protein (Figure 4A, Figure S1) and it was absent in the wt ILTV and ΔTK-GFP^+^ infected cell lysates.

We next assessed whether Cre–Lox mediated excision adversely impacted the inserted genes. Western blotting of the cells lysate, prepared from LMH cells which were infected with ΔTK&ΔUS4-dsRED^+^ ILTV as well as ΔTK&ΔUS4-Cre ILTV, showed expected expression of F protein and no corresponding expression was observed in cells which were infected with parental ILTV or recombinant ILTV lacking genes for F proteins of NDV (Figure 4B, Figure S1). These results demonstrate that Cre–Lox system can safely excise gene without negatively impacting the expression of associated proteins.

### 3.5. In Vitro Replication and Stability of Recombinant ILTVs

After assessing the successful expression of the F protein, we aimed to determine whether the insertion of the cassette influences the replication kinetics of the recombinant ILTVs. To this end, LMH cells were infected with parental and recombinant ILTV viruses and the virus replication rates were assessed using qPCR and fluorescent microscopy. Analysis of the genomic replication and viral replication progression (Figure 4C) did not show significant differences between recombinant ILTVs generated in this study and wild-type ILTVs.

In order to estimate the stability of the expression cassette, recombinant ILTV generated by genome editing were passaged for at least 15 times in LMH cells, and the stability of the inserted cassette was assessed using *F* gene specific PCR. Continuous assessment of the *F* gene cassette showed no apparent loss or reduction in the *F* gene products indicating a stable integration of the cassette in the ILTV genome (Figure 4D). Additionally, we infected the LMH cells with recombinant and parental ILTVs (passaged for at least 15 times) and measured the sizes of plagues. All viruses (recombinants and parental) showed insignificant variations in plagues sizes (Figure 4E).

Taken together, these data demonstrate that transgene insertion in the ILTV genome using CRISPR/Cas9 and excision of genes with Cre–Lox is a reliable approach to stably express genes.

## 4. Discussion

The ILTV vectors carry remarkable capacity to express heterologous antigens, and are consistently being applied in the poultry industry for immunisation against multiple viruses, including NDV, and influenza H5 and H7 strains [15,16,17]. With these proven applications of ILTV, the generation of recombinant viruses to expression foreign genes has been achieved using conventional recombination strategies, which are tedious, time-consuming, and error prone [18]. We have demonstrated here the feasibility of the CRISPR/Cas9 nucleases accompanied by the Cre–Lox recombinase in constructing a recombinant ILTV vector expressing either surface antigen of NDV or marker proteins (GFP and dsRED).

While ILTV proposes satisfactory induction of cellular and humoral immunity, and conferring protection against multiple strains of ILTV, latency, and reactivation are crucial for the permanence of ILTV in the poultry field. The molecular mechanisms in establishment and reactivation of ILTV latency are not fully understood. However, it has been proposed that the intimate viral–host interactions and the immune surveillance during latent infections are decisive factors [18]. Therefore, several previous studies have been conducted to delete virulence-determining gene such as dUTPase, thymidine kinase [10], secreted glycoprotein gG [35], tegument protein pUL47 [39], or iltovirus-specific pUL0 [7]. These gene-deleted ILTV mutants showed attenuation *in vivo*. However, these studies have only focused on the deletion of individual genes and thus limit its safety, and necessitating deletion of multiple non-essential genes to propose a better, safer and immune-competent vaccine vector.

This is the first study to demonstrate the application of CRISPR/Cas9 system in the development of recombinant ILTV vaccines. The presented pipeline proposes a straightforward, rapid, and efficient approach to develop ILTV-based novel recombinant vaccines to protect against major diseases of the poultry. Compared to traditional technologies for modification, the Cas9 endonuclease generates guided and targeted double stranded breaks, which are then repaired by either of the two cellular repair mechanisms; NHEJ or HDR [40,41]. In contrast to the HDR pathway, which occurs in the S and G2 phases of cell division, NHEJ is efficient and occurs throughout the cell division cycle [42]. Using single bait, red fluorescent protein (RFP) has been knocked-in to the pseudorabies virus with high efficacy through the NHEJ pathway [28]; however, this may lead to undesirable, error-prone, and unpredictable insertions. To mitigate this complication, we introduced dual-baits at the 5′ and 3′ ends of the insert. Since the bait sequence is independent of the sequence of the targeted virus and host, and is devoid of specific sgRNA selection, our benefactor system proposes a universal donor, which would be a valuable source for generation of multivalent vaccines against poultry pathogens.

As a proof of principle, we applied this approach to first generate a reporter ILTV by successfully and efficiently knocking-in a GFP expression cassette. In addition, we diversified the system by instantaneously deleting the virulence factors from the ILTV as a dynamic approach to concurrently attenuate the virus and to generate a marker virus. Using existing information on the roles of *TK* in ILTV attenuation [10], the generated ΔTK-GFP^+^ ILTV replicated competitively and sustainability. While individual genes have been deleted from ILTV genome, no studies have been performed to delete multiple genes from the same ILTV strain, which may propose a better and safer solution to the immunisation in poultry.

To follow up this line of safety, we generated a universal and repair donor vector system with a feature to efficiently excise dsRED marker flanked with LoxP sites and unique restriction sites to insert and swap heterologous genes of interest. We next applied our validated CRISPR/Cas9 approach to insert the entire cassette of ~5kb into the ILTV genome using gRNA flanking the *US4* virulence factor. The pipeline showed an efficient and rapid production of recombinant ILTV vaccine expressing heterologous antigen, fusion gene of NDV, and excisable dsRED. After successful rescue of the virus, *in vitro* characterisation and stability studies, the dsRED was deleted using Cre-recombinase enzyme. Insertion of the *F* gene expression cassette was stable and did not compromise the replication of ILTV, as was monitored until at least 15 passages *in vitro*. However, potential implications of inserted genes on the replication of recombinant ILTV “*in vivo”* and as “vaccine candidate” warrant further research. Moreover, this double genes-deleted recombinant ILTV will act as marker vaccine permitting differentiation of successfully immunised from infected chickens (DIVA). To facilitate the detection of TK-specific serum antibodies, competitive diagnostic assays can be devised utilising TK expression constructs and TK-specific monoclonal antibodies.

Taken together, we described a versatile and customisable pipeline for the development of NHEJ-CRISPR/Cas9 and Cre–Lox system in the development of innovative ILTV-vectored vaccines. While the approach has displayed the expression of the F protein of the NDV, it is feasible to develop future ILTV-vectored vaccines by insertion of multiple viral antigens at locations different than applied here. We have screened and identified additional sites, which appear safe and can accommodate longer genes without compromising the ILTV replication (Atasoy et al., unpublished data). Additionally, the same platform can be applied to engineer other avian DNA viruses to develop new multivalent-vectored vaccines for protecting multiple poultry diseases.

## 5. Conclusions

In summary, the application of CRISPR/Cas9 genome editing and Cre–Lox platforms are effective means to deploy new multivalent-vectored vaccines, which can be stepping stones to contain poultry infections, especially in disease-endemic countries where multiple pathogens circulate and pose devastating disease outcomes.

## Supplementary Materials

The following are available online at https://www.mdpi.com/2076-393X/7/4/207/s1: Figure S1. Raw figures where frames indicate the part of the figure used in actual manuscript; Table S1. Primers used in this study.
