# Supplementary material for: Simultaneous Deletion of Virulence Factors and Insertion of Antigens into the Infectious Laryngotracheitis Virus Using NHEJ-CRISPR/Cas9 and Cre–Lox System for Construction of a Stable Vaccine Vector
Source: Vaccines (Basel). 2019 Dec 5;7(4):207. doi: 10.3390/vaccines7040207 (PMC6963826; doi:10.3390/vaccines7040207)
Supplement: Supplementary file 1 [file vaccines-07-00207-s001.pdf]

Related to Figure 4

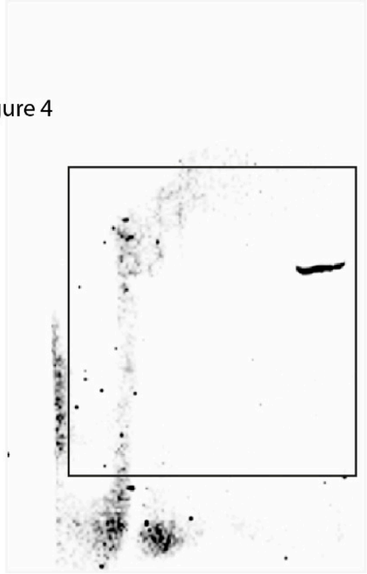

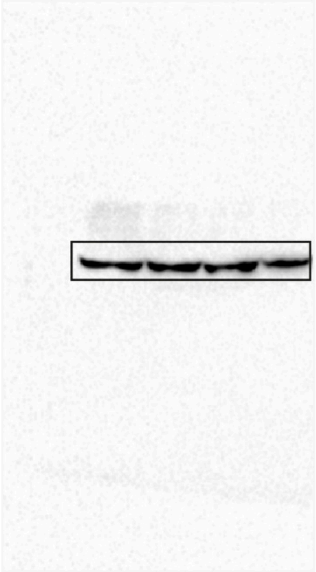

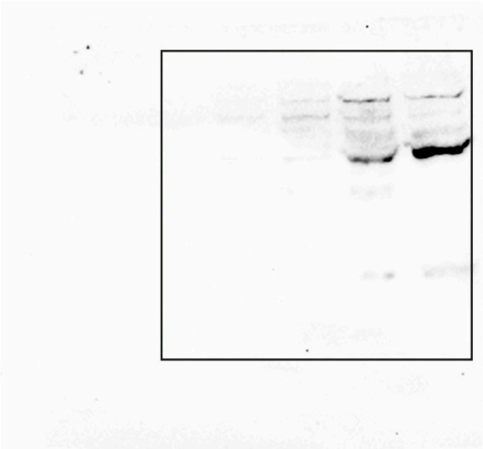

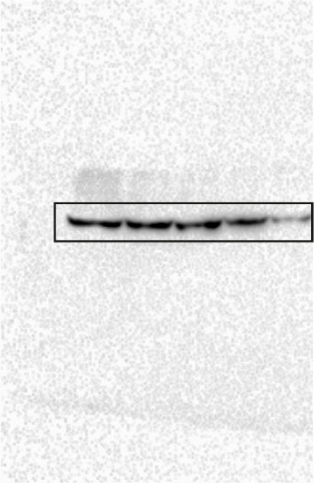

Related to Figure 4

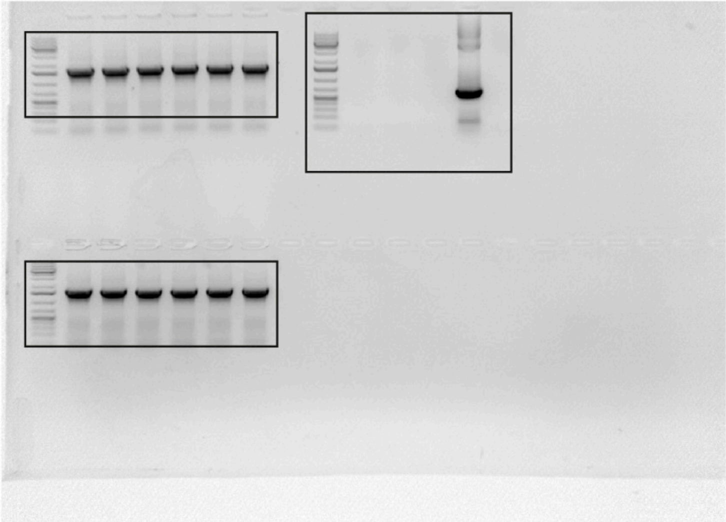

Table S1. Primers used in this study.

#	Primer	Sequence	Purpose	Reference
**1**	sg137152F	CACCGTAGCCTCGCCCGAACTCGA	For cutting UL4 gene region	This study
	sg137152R	AAACTCGAGTTCGGGCGAGGCTAC		
**2**	sg136416F	CACCGTCAGCGGGTACACTTTATA	For cutting UL47/US4 intergenic region	This study
	sg136416R	AAACTATAAAGTGTACCCGCTGAC		
**3**	sgA-Fwd	CACCGAGATCGAGTGCCGCATCAC	For releasing expression casettes from the donor plasmid	10.1093/nar/gkw064
	sgA-Rev	AAACGTGATGCGGCACTCGATCTC		
**4**	sgB-Fwd	CACCGGCAGCCGAAGCAACATGGG	For releasing expression casette of F gene (MCS1)	10.1242/dev.099085
	sgB-Rev	AAACCCCATGTTGCTTCGGCTGCC		
**5**	dsREDClon2F	CCGGAATTCTCCACCATGGCCTCCTCCGAGGACG	mRFP gene amplification primers for MCS2	This Study
	dsREDClon2R	GTACTCTAGAGGCGCCGGTGGAGTGGC		
**6**	FClon1F	CTAGCTAGCTCCACCATGGGCTCCAAACTTTCTA	F gene amplification primers for MCS1	This Study
	FClon1R	GCCGGTACCTGCTCTTGTAGTGGCTCT		
**7**	CMV-MCS1-F	AGAACCCACTGCTTACTGGCTT	Internal control primer set for MCS1	This Study
	CMV-MCS1-R	AACTAGAAGGCACAGTCGAGGC		
**8**	pEF-MCS2-F	TCAAGCCTCAGACAGTGGTTCA	Internal control primer set for MCS2	This Study
	pEF-MCS2-R	TTCCGCCTCAGAAGCCATAGAG		
**9**	IR/US4F	TTAAGCCGGTGATAGACGAGCC	Primer set for correction of recombinant virus	This Study
	IR/US4R	TTAGCACAGACACGCAAACCAC		
